# Pre-exposure prophylaxis as a complementary vaccination strategy to reduce rabies in Kerala, India: a cost-effectiveness modelling study

**DOI:** 10.1016/j.lansea.2026.100854

**Published:** 2026-09-05

**Authors:** Martha M. Luka, Zinia Thajudeen Nujum, Miqdad Asaria, Elaine A. Ferguson, Sivanandan Harikumar, Daniel T. Haydon, Rony Ray John, Katie Hampson

**Affiliations:** a School of Biodiversity, One Health & Veterinary Medicine, College of Medical, Veterinary and Life Sciences, University of Glasgow, Glasgow, G12 8QQ, Scotland, UK; b Department of Community Medicine, Government Medical College, Kollam, Kerala, India; c School of Public Health, Kerala University of Health Sciences, Kerala, India; d Department of Health Policy, London School of Economics and Political Science, London, UK; e REAL Centre, The Health Foundation, London, UK; f Directorate of Health Services, Kerala, India; g Directorate of Animal Husbandry, Kerala, India

**Keywords:** One health, Neglected tropical diseases, *Lyssavirus*, Elimination, Decision tree

## Abstract

**Background:**

Post-exposure prophylaxis (PEP) and dog vaccination continue to be the widely adopted vaccination strategy for rabies control in high-burden countries like India. To determine the public health and economic impacts of childhood pre-exposure prophylaxis (PrEP) relative to existing strategies, we carried out a modelling analysis in the state of Kerala, India.

**Methods:**

We adapted a probabilistic decision-tree model over a 10-year time horizon (2026–2035). This timeframe is sufficient for dog vaccination to interrupt rabies transmission and for protection to accrue from PrEP delivered through routine childhood immunisation. The model simulates interactions between dog vaccination, PrEP, and PEP to estimate health provider costs, quality-adjusted life years (QALYs) gained, and net health benefit across a range of willingness-to-pay thresholds.

**Findings:**

Assuming current practice of four-dose intradermal PEP and sub-optimal dog vaccination, we projected 431 rabies deaths (95% Prediction interval [PI] 353–523) and a cost of US$50.3 million over ten years, with dog vaccination responsible for almost 70% of deaths averted by this strategy. Routine childhood PrEP increased costs by US$10.5–36.5 million (INR 1.0–3.4 billion), depending on the regimen used, and cumulatively prevented only ten additional deaths beyond those averted by current practice. Incorporating PrEP was not optimal (probability ≤12%) across India's willingness-to-pay threshold. The intensified owned-dog vaccination strategy achieved the highest benefit at India's mean willingness-to-pay (739,420 QALYs; 28% probability of optimality), while adopting WHO's recommended abridged PEP regimen was the only cost-saving strategy (US$6.3 million saved; equivalent mortality to status quo). Comprehensive dog vaccination coverage of 70% combined with abridged PEP regimens became optimal at India's upper willingness-to-pay threshold, reducing deaths by 83% compared to current practice.

**Interpretation:**

In high-PEP-access settings, PrEP is not cost-effective and provides negligible benefit. To maximise lives saved, PrEP should only be considered once gains from strengthening dog vaccination and PEP delivery have been exhausted.

**Funding:**

Wellcome Trust; Indian Council of Medical Research - Department of Health Research (ICMR-DHR) International Fellowship for Senior Biomedical Scientists.

**Research in context**

**Evidence before this study**

Rabies pre-exposure prophylaxis (PrEP) is a biologically plausible complement to post-exposure prophylaxis (PEP), but its programmatic value remains debated. We searched PubMed using the terms ***“rabies” AND “cost” AND (“PrEP” OR “pre-exposure”)*** for articles published between January 1, 2000 and July 10, 2026. The search identified 65 records, including six primary economic evaluations conducted in endemic settings and two systematic reviews. Findings were varied and context dependent. Three studies concluded that PrEP is not cost-effective or cost-saving under most real-world conditions, and that mass dog vaccination is more efficient in reducing population-level burden. The favourable analyses each identified specific enabling conditions: high bite incidence (>2000 per 100,000), high rabies positivity among biting animals (>15%), constrained PEP access, or low vaccine costs (<$5 per dose). However, these conditions lack empirical precedent, and no studies evaluated PrEP in settings with high PEP access, leaving uncertainty about its incremental value.

**Added value of this study**

This study evaluates routine childhood PrEP and alternative prevention strategies in Kerala, where PEP access is high, yet rabies deaths persist, to quantify the incremental benefit PrEP can provide. Strategies combining PrEP, PEP, and dog vaccination were systematically compared to define the efficiency frontier for ending rabies deaths. Routine childhood PrEP was found to prevent very few additional deaths (approximately ten over a ten-year time horizon) compared with current practice, despite increasing programme costs by over US$10 million. Overall, PrEP had a low probability of being the optimal strategy across India's willingness-to-pay range. In contrast, strengthening dog vaccination produced substantially larger reductions in mortality, with high vaccination coverage reducing annual deaths to near zero within approximately three years. Additionally, adopting WHO's abridged PEP regimen was cost-saving without increasing mortality.

**Implications of all the available evidence**

Previous studies suggest that PrEP is most valuable where exposure is high, PEP access is limited, or vaccine costs are low. Our findings show little incremental benefit where PEP access is high. Persistent rabies deaths in such settings do not justify population-wide PrEP. Greater health gains are likely from sustained dog vaccination and addressing remaining gaps in timely PEP access, while use of dose-sparing PEP regimens can reduce programme costs. Progress towards Zero by 30 should therefore prioritise dog vaccination and equitable PEP delivery. Targeted PrEP may be justified for high-risk populations but should complement rather than displace investment in these core interventions.

## Introduction

Globally, dog-mediated rabies remains a persistent public health problem, causing an estimated 59,000 deaths annually,^1^ and India bears the largest burden at approximately 5700 deaths.^2^ Two complementary prevention strategies underpin WHO's ‘Zero by 30’ goal: mass dog vaccination to interrupt transmission at source and post-exposure prophylaxis (PEP) comprising anti-rabies vaccine and rabies immunoglobulins for severe exposures.^3^ Despite their efficacy, both strategies face implementation challenges. Dog vaccination coverage remains insufficient in endemic regions due to logistical barriers, insufficient political commitment, and competing priorities,^4^ despite its potential to eliminate exposures, reduce PEP demand and relieve pressure on the health systems. Meanwhile, PEP access is undermined by supply chain failures, geographic barriers, and low healthcare-seeking.^5^

The state of Kerala in India provides PEP free of cost to more than 900,000 people annually, with PEP completion rates exceeding 90%,^6^ far above the national average of 57–66%.^2^^,^^7^ Yet Kerala reported 5 to 33 rabies deaths annually between 2012 and 2025.^6^ Similarly, in the Philippines, expanding PEP access initially decreased rabies deaths, but subsequently bite-patient incidence increased from 200 to over 1000 per 100,000 population annually, without further mortality reductions.^8^ These statistics suggest a subset of exposed individuals do not recognise their risk, face residual barriers to care, or present late, raising the question of what is needed to prevent these rabies deaths.

Pre-exposure prophylaxis (PrEP) primes the immune system before rabies exposure,^9^^,^^10^ reducing the doses required for post-exposure vaccination and eliminating the need for immunoglobulins.^3^ PrEP may also provide protection when PEP is delayed, incomplete, or not sought.^9^ However, the economic case for PrEP in endemic settings is contested.^11^^,^^12^ Cost-effectiveness varies substantially with the incidence of bite patient presentations, rabies exposures amongst these patients, vaccine prices, and PEP access.^13^, ^14^, ^15^, ^16^ WHO-commissioned work cautions that PrEP is unlikely to be efficient in most endemic settings compared with optimised PEP and dog vaccination.^17^

In the state of Kerala, where access to PEP and completion rates are high, the additional benefit of PrEP beyond existing PEP provision remains uncertain, particularly for under-reached populations, including geographically dispersed tribal communities (∼1.5% of the population) who face documented barriers to care.^18^ The central question is whether PrEP's additional protection justifies its integration into routine immunisation to represent a cost-effective complement to existing prevention strategies and accelerate the progress towards zero human deaths due to rabies. In this study we evaluate the cost-effectiveness of routine childhood PrEP from the health provider perspective. Specifically, we assess whether PrEP provides meaningful reductions in mortality and explore outcomes if PrEP investment displaces funding for dog vaccination, comparing the cost-effectiveness of alternative strategies.

## Methods

**Fig. 1:**
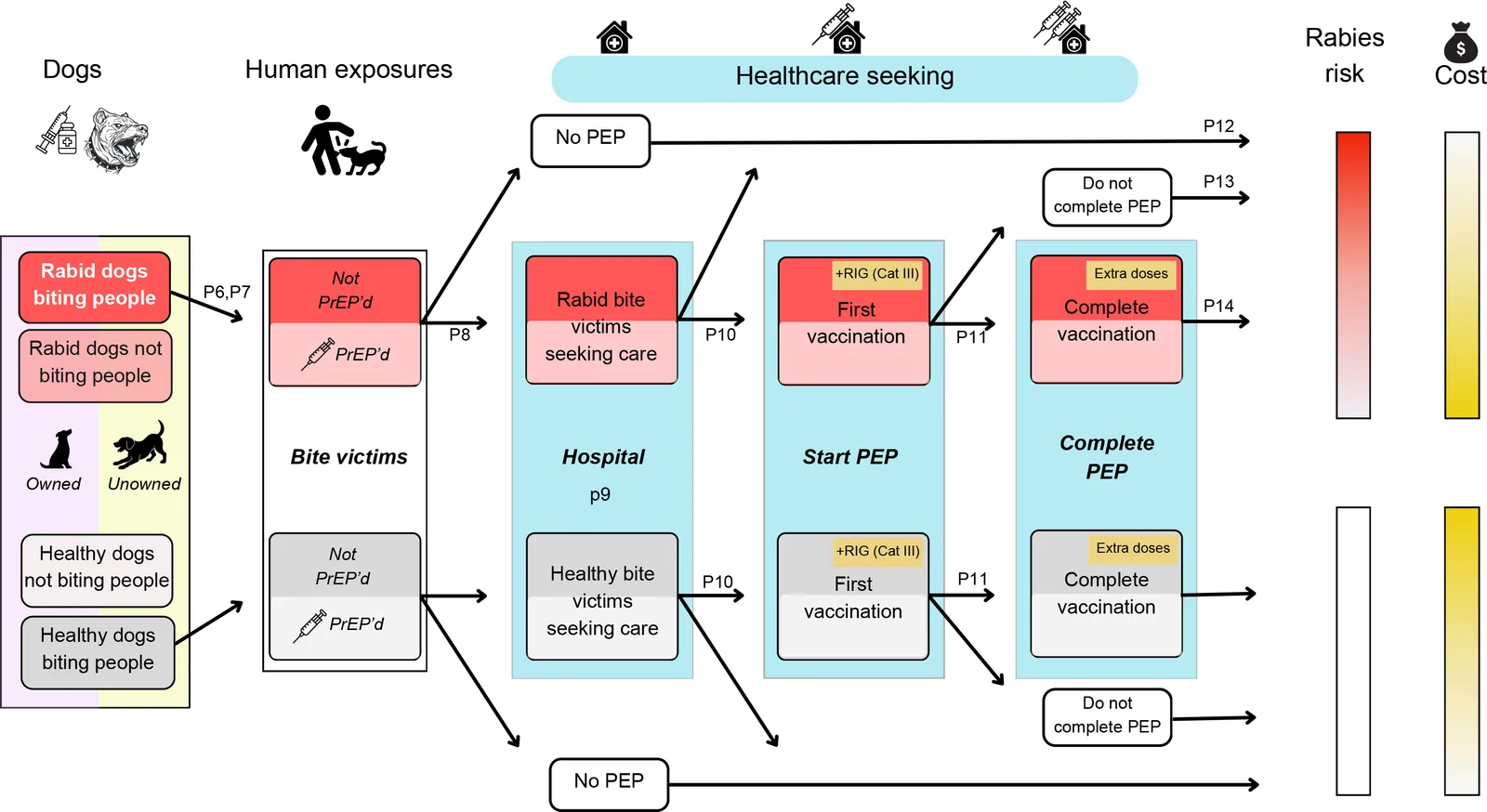
**Conceptual framework of the decision tree model for rabies control and prevention.** The model simulates rabies transmission and prevention pathways from dog infection to human health outcomes and costs. Dogs are stratified into owned and unowned subgroups, with differential vaccination coverage determining rabies incidence. Following bites from rabid or healthy dogs, victims' prior PrEP status influences subsequent care pathways. Healthcare-seeking, PEP initiation, and regimen completion are modelled probabilistically. Rabies risk (red gradient) decreases with complete PEP; programme costs (yellow gradient) accumulate with vaccine doses, RIG administration, and clinic visits. Numbered arrows indicate the sequence of transitions modelled in the decision tree, with respective parameters detailed in Supplementary Table S1.

We adapted a probability-based decision-tree model^19^ to evaluate the public health and economic impact of rabies prevention strategies in Kerala, India over a 10-year time horizon (2026–2035). This timeframe is sufficient for dog vaccination to interrupt rabies transmission and for protection to accrue from PrEP delivered through routine childhood immunisation. The model simulates interactions between dog vaccination, PrEP, and PEP (Fig. 1), capturing dog rabies incidence as a function of vaccination coverage; rabid dog bite exposures and healthcare-seeking; PrEP and PEP uptake, adherence and effectiveness; and health and economic outcomes. We ran 3000 stochastic realisations per scenario, summarising rabies exposures, deaths, deaths averted relative to a zero-intervention counterfactual, biologicals consumed, and total costs as medians and 95% percentile intervals.

Dogs were stratified into owned and unowned (stray/free roaming) subgroups to capture differential vaccination accessibility and costs. Owned dogs are vaccinated through veterinary clinics, and unowned dogs through catch-vaccinate-release strategies at higher per-dog costs. Annual dog rabies incidence was modelled as a function of vaccination coverage and the previous year's incidence using generalised linear models fitted to Tanzanian longitudinal data.^20^ This dataset was selected as it represents the most detailed available source capturing the empirical relationship between vaccination coverage and subsequent rabies incidence in dogs. Because these models were fitted to observed field outcomes rather than vaccine efficacy studies, the estimated effects of vaccination represent realised programme effectiveness under operational conditions and implicitly account for imperfect vaccine delivery and other sources of reduced efficacy.^21^ Rabies was assumed to circulate freely among owned and unowned dogs, and an upper incidence was introduced to prevent unrealistically high simulated incidences in periods of low vaccination, Supplementary Table S1. Uncertainty was accounted for by drawing a new set of model parameters from the posterior distribution for each realisation. We divided Kerala's dog population into 22 independent simulation units to match the Tanzanian dog population parameters which is used to generate the model, and results were aggregated. Human bite exposures per rabid dog were modelled using a negative binomial distribution,^20^ assuming equal biting propensity regardless of ownership status. Bites from healthy dogs were estimated by subtracting predicted rabid-dog exposures from total bite presentations recorded in surveillance.

### Decision tree model

Dog bites trigger a sequence of probabilistic steps (Fig. 1) determined by PrEP status, healthcare-seeking, and PEP administration. PrEP scenarios were modelled as a one-time school-based campaign (among ages 5–15 years), followed by routine infant immunisation in subsequent years, with coverage equal to Kerala's routine immunisation (Supplementary Table S1). We assumed PEP completion requires at least three doses for PrEP-naive individuals and two doses for PrEP recipients.^3^

Although laboratory and limited clinical evidence suggest PrEP may protect against rabies without PEP,^9^ WHO recommends PEP following all exposures regardless of PrEP status.^22^ We therefore assume that PrEP does not prevent rabies in our base case (PrEP effectiveness = 0), with benefit arising solely from the reduced post-exposure doses required for PrEP-primed individuals, improving outcomes among those with suboptimal adherence to PEP (Supplementary Table S1). Standalone PrEP protection was explored in sensitivity analyses.

We estimated health provider costs reflecting Kerala's policy of providing free of cost PEP and under the assumption that PrEP would also be provided free of cost. Costs included vaccines, syringes, and administration, but excluded broader health system costs and cost recovery for owned-dog vaccination. Vaccine wastage from the discard of partially used vials was set at 10%, consistent with efficient vial use during intradermal administration in high-throughput clinics.^6^ Costs were calculated in 2026 US dollars (92 INR/US$).

Quality-adjusted life years (QALYs) gained were estimated as the difference between Kerala's life expectancy and the mean age at bite, applying a utility weight of 1.0, reflecting that symptomatic rabies is invariably fatal, with a negligibly short period of ill health before death. Both costs and QALYs were discounted at a common annual rate of 3%^23^ over the 10-year horizon.

Cost-effectiveness was analysed using the net health benefit (NHB) framework across (willingness-to-pay) WTP thresholds from 0 to 5000 US$ per QALY. This range covers India's current WTP, estimated at US$2535 per QALY (range US$2034–3092),^24^ equivalent to 0.94 × GDP per capita, based on recent World Bank estimates. NHB was estimated for all scenarios, and cost-effectiveness acceptability curves were used to identify the probability that each strategy maximises population health across the WTP range. The strategy with the highest NHB at each threshold was considered optimal. Decision uncertainty was characterised by the spread across the 3000 stochastic realisations, propagating uncertainty simultaneously across all model inputs.

### Scenarios analysed

We evaluated 12 scenarios with varying PrEP and PEP regimens, and mass dog vaccination (MDV) coverage in owned and unowned dogs. These are summarised in Table 1. Scenario A (status quo) reflects current practice, with no routine PrEP, use of India's four-dose intradermal (ID) PEP with immunoglobulin for Category III exposures, and sub-optimal MDV coverage (3% coverage of unowned dogs and 50% coverage of owned dogs). Scenarios B–D remain with status quo MDV suboptimal coverage but introduced childhood PrEP under three PEP regimens: India's four-dose PEP and three-dose ID PrEP schedule (B), and WHO's one-week PEP with single-dose intramuscular PrEP (C) or two-dose intradermal PrEP (D). In these PrEP scenarios, recipients received an abbreviated two-dose post-exposure regimen without immunoglobulins. Scenarios E and F examine PrEP-MDV interactions: resource diversion from dog vaccination (E, unowned dog vaccination coverage dropping to 0%) and simultaneous PrEP and unowned-dog MDV scale-up (F, 70%). Scenarios G–J do not include PrEP and focus on increasing MDV coverage of unowned dogs (G = 30%, H = 70%), owned dogs (I = 80%), or both unowned and owned dogs (J, both at 70%), establishing a benchmark for PrEP comparisons. Scenarios K and L assess WHO's one-week PEP schedule under status quo and scaled-up MDV coverage (70% coverage of both owned and unowned dogs), respectively.

**Table 1:** Intervention scenarios evaluated.

Scenario	PrEP	PEP	Unowned dogs MDV coverage	Owned dogs MDV coverage
A. SQ	none	4-dose ID, RIG for cat III bites (38% of bite patients)	3%	50%
B. SQ + PrEP (India regimen)	3-dose ID	No PrEP: 4-dose ID & RIG (as per A) PrEP: 2-dose PEP (& no RIG for cat III)	3%	50%
C. WHO PEP + 1-shot PrEP	1-dose IM	No PrEP: 1-week ID & RIG PrEP: 2-dose PEP (& no RIG for cat III)	3%	50%
D. WHO PEP & PrEP	2-dose ID	No PrEP: 1-week ID & RIG PrEP: 2-dose PEP (& no RIG for cat III)	3%	50%
E. D (WHO PEP & PrEP) + drop in MDV coverage	2-dose ID	No PrEP: 1-week ID & RIG PrEP: 2-dose PEP (& no RIG for cat III)	0%	50%
F. D (WHO PEP & PrEP) + MDV scale-up in unowned dogs	2-dose ID	No PrEP: 1-week ID & RIG PrEP: 2-dose PEP (& no RIG for cat III)	70%	50%
G. SQ + moderate MDV scale-up in unowned dogs	none	4-dose ID, RIG for cat III bites	30%	50%
H. SQ + MDV scale-up in unowned dogs	none	4-dose ID, RIG for cat III bites	70%	50%
I. SQ + MDV intense scale-up in owned dogs	none	4-dose ID, RIG for cat III bites	3%	80%
J. SQ + MDV scale-up in owned and unowned dogs	none	4-dose ID, RIG for cat III bites	70%	70%
K. WHO PEP + SQ MDV	none	1-week ID, RIG for cat III bites	3%	50%
L. WHO PEP + MDV scale-up in owned and unowned dogs	none	1-week ID, RIG for cat III bites	70%	70%

Abbreviations: SQ = status quo, PrEP = pre-exposure prophylaxis, PEP = post-exposure prophylaxis, RIG = Rabies Immunoglobulin, IM = Intramuscular, ID = Intradermal, MDV = Mass dog vaccination, cat = WHO exposure category.

PrEP regimens: *3-dose ID (Indian)* = 1 × 0.1 ml on d0,7,21/28; *1-shot IM* = 1 ml; 2-dose ID = 2 × 0.1 ml on d0&7. PEP regimens: *4-dose ID* = 2 × 0.1 ml on d0, 3, 7, 28; *1-week ID* = 2 × 0.1 ml on d0, 3 & 7.

### Statistical analysis

Parameters (Supplementary Table S1) were derived from Kerala, where available, supplemented by India-specific estimates and Tanzanian data.^5^^,^^6^^,^^20^ Healthcare-seeking, PEP initiation, and adherence were parameterised from hospital surveillance and a pilot of Integrated Bite Case Management (IBCM) in Kerala. PEP adherence was modelled as geometric decay calibrated to >90% completion of at least three doses (per-dose probability 96.5%).

The 2019 livestock census reported 836,000 owned and 289,000 unowned dogs (human-to-dog ratio 31:1), but unowned populations are frequently undercounted several-fold.^25^ We calibrated the model by jointly varying the unowned dog multiplier (×1 − ×7) and healthcare-seeking probability (70–100%), fitting against annual reported rabies deaths (5–33) and the proportion of rabies exposures among bite patients (0.5–3%), using a composite least-squares loss function that equally weighted both targets. The parameters that best fit both indicators were selected as the base case. We accounted for underreporting,^26^ recognising that national surveillance captures as little as 1–10% of rabies deaths,^2^ whereas Kerala's stronger surveillance likely results in higher ascertainment (50–70%).

We systematically varied four parameters alongside PrEP coverage: healthcare-seeking (50–95%); standalone PrEP effectiveness (0–100%); unowned dog vaccination coverage (0–70%); and the unowned dog population multiplier (one- to fivefold).

### Role of the funding source

Funders had no role in the study design, data collection, analysis, interpretation, writing, or publication decisions.

## Results

The best-fitting parameter combination from model calibration identified a healthcare-seeking probability of 95% for rabies exposures and an unowned dog population five times the census estimate, yielding a human-to-dog ratio of 15.3:1 (2.3 million dogs, 63% unowned). Under this parameterisation, the model reproduced observed surveillance measures, predicting 43 rabies deaths annually (95% PI: 35–51, 50–70% recorded in surveillance) and an exposure proportion of 0.52% of bite patients (95% PI: 0.44–0.61%), Supplementary Figure S1.

**Fig. 2:**
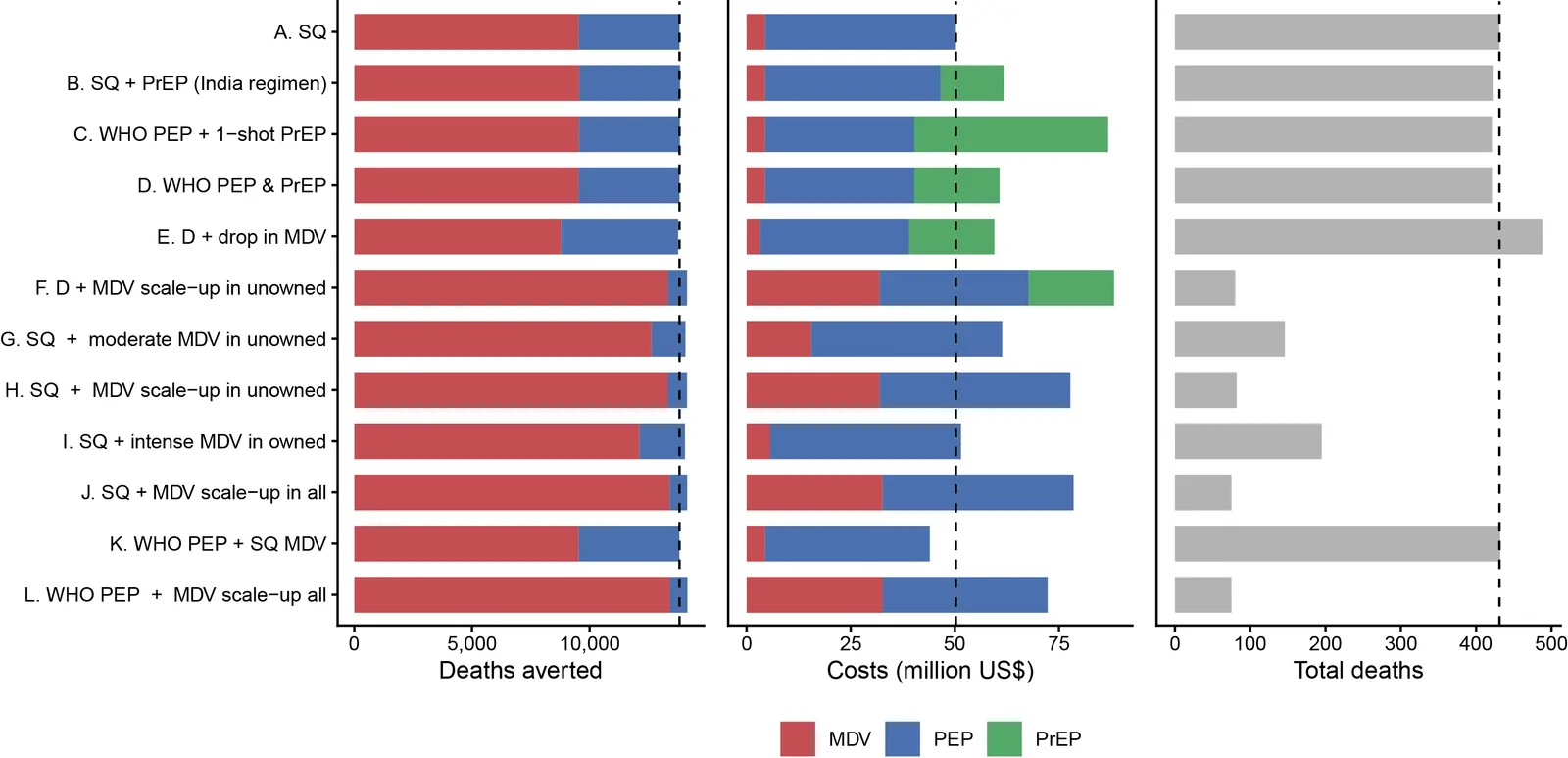
**Health impact and programme costs across rabies intervention scenarios in Kerala, India (2026**–**2035).** Horizontal bars show (left) total rabies deaths averted relative to a zero-intervention counterfactual, (middle) total programme costs over 10 years (million US$), disaggregated by intervention component: mass dog vaccination (MDV), post-exposure prophylaxis (PEP), and pre-exposure prophylaxis (PrEP); and (right) total rabies deaths over the 10-year horizon. Scenarios vary PEP and PrEP regimens, and MDV coverage in owned and unowned dogs. Vertical dashed lines indicate the corresponding values under the status quo (SQ) scenario, providing a reference for comparison across strategies.

Over the 10-year time horizon (2026–2035), the status quo scenario (A) resulted in 431 human rabies deaths (95% PI: 353–523) and incurred a total cost of US$50.3 million (4.6 billion INR). PEP accounted for 91% of programme costs (US$45.8 million), while dog vaccination averted 69% of deaths (9528 of 13,784), Fig. 2. The sole cost-saving strategy was adoption of WHO's abridged PEP regimen at status quo dog vaccination coverage (Scenario K), reducing total costs to US$44.0 million with no change in rabies deaths.

Routine childhood PrEP, delivered via a one-time school-based campaign, achieved 14% population coverage, with annual infant immunisation incrementally increasing coverage thereafter. PrEP scenarios (B–D) reduced cumulative deaths by fewer than ten relative to the status quo at additional costs of US$10.5–36.5 million. The scenario whereby PrEP diverts funds from dog vaccination and reduces coverage in unowned dogs (Scenario E) was the worst-performing strategy, increasing deaths to 488 (95% PI: 401–581) with costs 18% higher than the status quo, Table 2 and Fig. 2. Annual exposure and mortality trajectories under PrEP scenarios were largely overlapping and did not approach zero deaths (Supplementary Figure S2). Even under optimistic assumptions of full standalone PrEP effectiveness, a maximum of 79 incremental deaths were averted across PrEP scenarios, well below the 238–357 deaths averted by dog vaccination-focused strategies at comparable or lower cost (Supplementary Figure S3).

**Table 2:** Health outcomes and cost-effectiveness of rabies prevention scenarios in Kerala, India (10-year horizon, 2026–2035).

Scenario	Deaths	Deaths averted	QALYs gained (discounted)	Total costs ($M)	Mean NHB at WTP = US$2535	Probability optimal	Rank
A. SQ	431 (353–523)	13,784 (12,840–14,732)	747,238 (696,439–799,226)	50.26 (49.90–50.64)	727,364	6%	8
B. SQ + PrEP (India regimen)	422 (341–509)	13,805 (12,823–14,756)	748,260 (695,302–799,765)	61.88 (61.50–62.25)	723,591	4%	10
C. WHO PEP + 1-shot PrEP	421 (344–506)	13,802 (12,854–14,729)	747,961 (696,503–798,061)	86.79 (86.41–87.16)	713,672	2%	12
D. WHO PEP & PrEP	421 (343–505)	13,782 (12,866–14,734)	747,188 (697,231–798,146)	60.73 (60.37–61.11)	723,749	5%	9
E. D + drop in MDV coverage	488 (401–581)	13,714 (12,797–14,692)	743,769 (693,825–796,800)	59.49 (59.10–59.86)	720,876	4%	11
F. D + MDV scale-up (unowned dogs)	80 (58–107)	14,141 (13,149–15,094)	766,168 (711,909–818,484)	88.23 (86.08–92.51)	731,103	9%	6
G. SQ + moderate MDV (unowned dogs)	146 (110–187)	14,058 (13,101–15,043)	761,957 (709,504–815,429)	61.37 (60.33–63.23)	737,967	13%	3
H. SQ + MDV scale-up (unowned dogs)	82 (59–109)	14,144 (13,150–15,076)	766,428 (712,366–817,177)	77.75 (75.59–82.16)	734,852	11%	4
I. SQ + intense MDV (owned dogs)	195 (149–250)	14,028 (13,054–14,946)	760,025 (707,119–810,827)	51.48 (50.95–52.02)	739,420	14%	1
J. SQ + MDV scale-up (all dogs)	75 (53–103)	14,140 (13,187–15,077)	765,993 (714,355–817,240)	78.50 (76.31–83.07)	734,653	10%	5
K. WHO PEP + SQ MDV	432 (353–519)	13,774 (12,811–14,736)	746,844 (694,224–799,054)	44.00 (43.62–44.37)	729,248	7%	7
L. WHO PEP + MDV scale-up (all dogs)	75 (53–100)	14,149 (13,169–15,152)	766,558 (713,364–821,356)	72.28 (70.19–76.76)	738,111	14%	2

Values are medians (95% percentile intervals). Deaths averted are relative to a zero-intervention counterfactual. The status quo (SQ) reflects current practice (A): no Pre-Exposure Prophylaxis (PrEP), four-dose intradermal post-exposure prophylaxis (PEP) with RIG for category III exposures, and sub-optimal mass dog vaccination (MDV) coverage (3% unowned, 50% owned dogs). Scenarios B–F introduce childhood PrEP under varying PEP and MDV configurations. Scenarios G–J evaluate MDV scale-up without PrEP. Scenarios K and L assess WHO's abridged one-week PEP regimen. Full scenario specifications are in Table 1. NHB = net health benefit; QALYs = quality-adjusted life years; WTP = Willingness to pay.

Scaled-up dog vaccination produced substantially greater mortality reductions across all configurations, with deaths approaching near-zero within three years under higher coverage scenarios (Supplementary Figure S2). Intensifying owned-dog vaccination alone (Scenario I) reduced deaths to 195 (95% PI: 149–250) at US$51.5 million, the lowest cost among strategies achieving meaningful mortality reduction. Scaling unowned-dog vaccination to 70% coverage (Scenario H) reduced deaths to 82 (95% PI: 59–109). Combining comprehensive dog vaccination with WHO's abridged PEP regimen (Scenario L) achieved equivalent mortality reduction at US$72.3 million. Adding PrEP to scaled-up unowned dog vaccination (Scenario F) increased costs to US$88.2 million with no meaningful reduction in deaths relative to dog vaccination alone (Scenario H), Table 2.

Scenarios differing primarily in PEP or PrEP regimen yielded near-identical efficiency (Supplementary Figure S2). The full set of 12 scenarios was evaluated to explore the intervention design space and identify the most informative policy alternatives. Because several scenarios represent variations of the same underlying strategy rather than mutually exclusive alternatives, subsequent cost-effectiveness analyses focused on five scenarios: the status quo (A), the most feasible PrEP strategy under current Indian guidelines (B), and the three strategies that define the efficiency frontier (K, I, L). Full results for all 12 scenarios are presented in Supplementary Table S2, whereas the probability-of-optimality estimates reported below are conditional on the reduced five-scenario comparison.

**Fig. 3:**
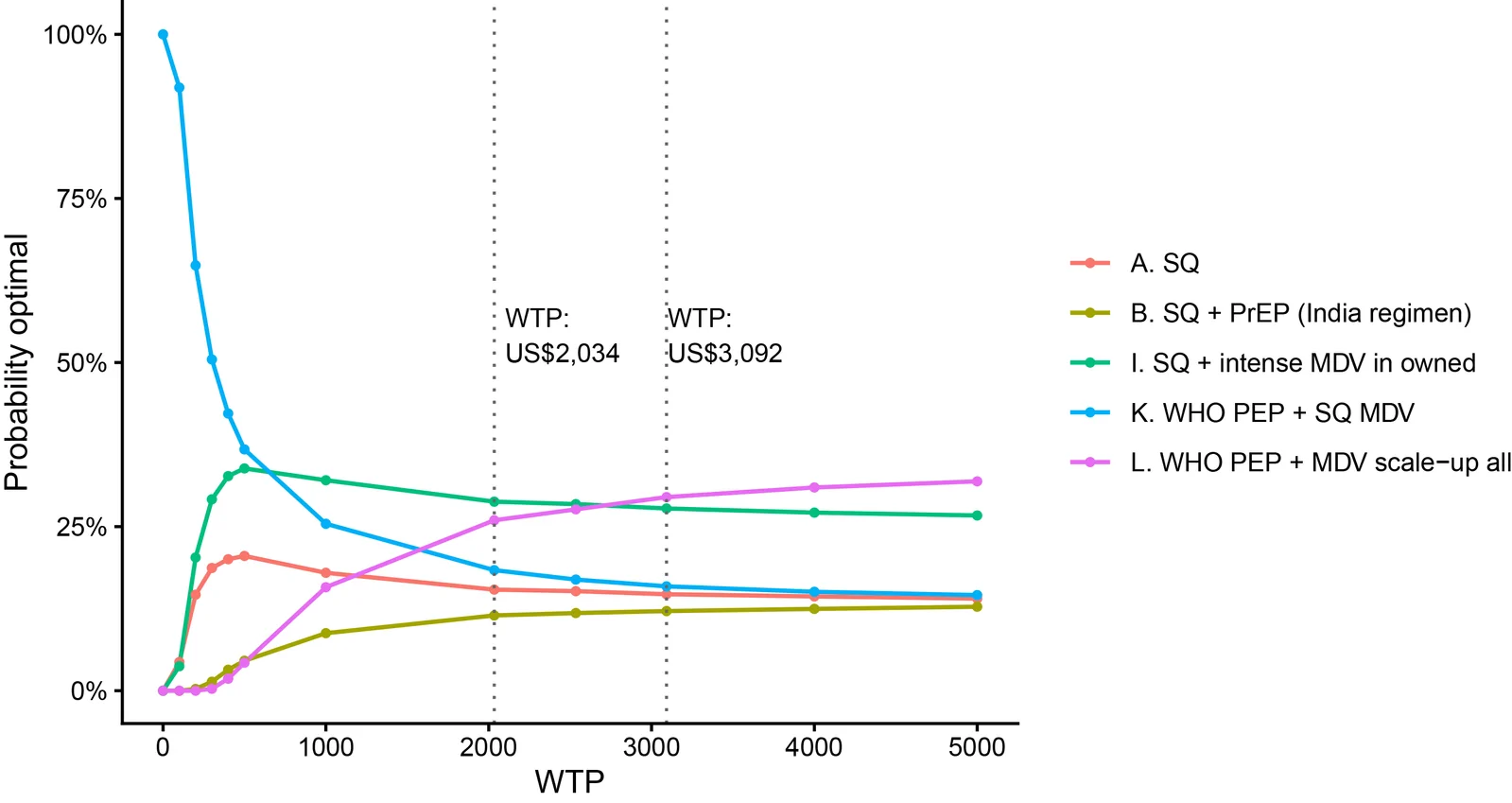
**Cost-effectiveness acceptability curves.** Curves show the probabilities that a strategy is optimal across a range of willingness-to-pay (WTP) thresholds. Vertical dashed lines indicate lower and upper bounds for India's WTP (US$2034 and US$3092 per QALY gained, respectively). All costs are reported in 2026 US$. QALYs = quality-adjusted life-years. GDP = gross domestic product. WTP = willingness to pay.

Results across the WTP range follow a coherent, interpretable pattern (Supplementary Table S3, Fig. 3). At low thresholds, Scenario K (changing PEP from the current Indian regimen to the WHO-recommended one-week regimen), which is cost-saving and requires no new infrastructure, was optimal. This strategy had a 65% probability of optimality at US$200 per QALY. As the WTP threshold approached India's estimated range (US$2034–3092 per QALY), strategies focused on dog vaccination that avert more deaths at higher cost became preferable: Scenario I (intensive vaccination of owned dogs to achieve 80% coverage) led at the lower and mean threshold (NHB 734,419 and 739,420 QALYs, respectively), while Scenario L (70% coverage of both owned and unowned dogs) progressively closed the gap, matching and exceeding Scenario I at the upper bound (743,281 vs 743,078 QALYs). This crossover reflects the point at which the additional mortality gains from vaccinating unowned dogs justify their higher operational costs. Should Kerala's health system move toward a higher societal valuation of health gains (WTP of US$5000 per QALY), Scenario L would become the clear optimal strategy (752,261 QALYs; 32% probability of optimality). Across India's full WTP range, PrEP (Scenario B) yielded a lower NHB than the status quo, with the probability of optimality not exceeding 12%.

Reducing the human vaccine vial price for routine PrEP to 50% or 25% of the base-case value increased its probability of optimality but did not make PrEP the preferred strategy across India's willingness-to-pay range; dog vaccination and WHO PEP strategies remained more likely to be optimal, Supplementary Figure S4.

### Sensitivity analysis

**Fig. 4:**
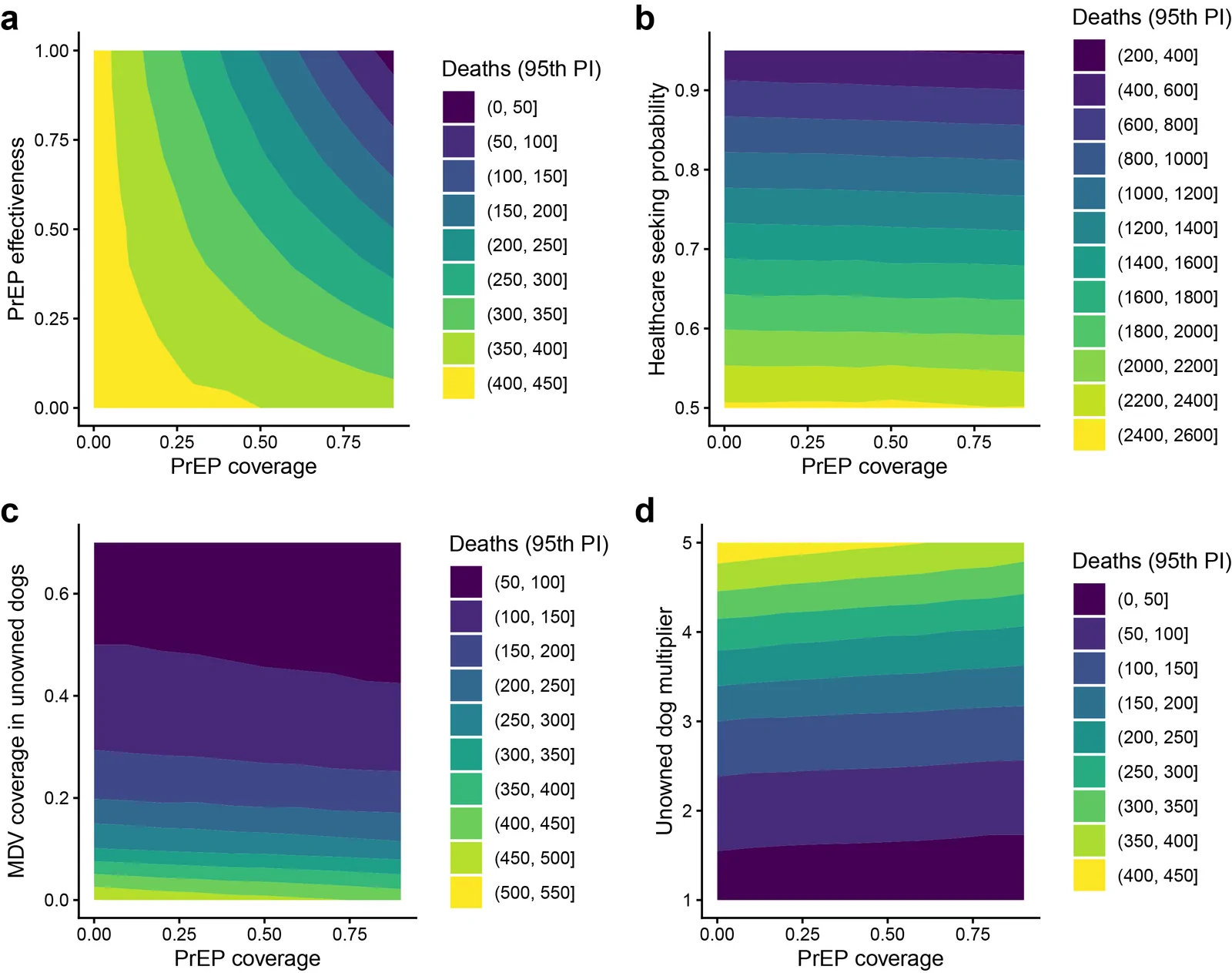
**Sensitivity of projected rabies deaths to PrEP coverage and other key parameters (2026**–**2035).** Contour plots show projected rabies deaths (median of model simulations) over 10 years under varying assumptions. (A) Joint effects of PrEP coverage and PrEP effectiveness. (B) Interaction between PrEP coverage and the probability of healthcare-seeking following exposure. (C) Interaction between PrEP coverage and MDV coverage among unowned dogs. (D) Interaction between PrEP coverage and the assumed multiplier applied to the unowned dog population. Colours indicate increasing numbers of deaths (yellow = higher; dark purple = lower).

Under the base-case assumption of zero standalone PrEP effectiveness, deaths declined from 431 with no PrEP to 379 at 90% PrEP coverage, a 12% reduction. Under base-case PrEP coverage (14% by the end of Year 1), deaths were reduced by less than 2%. A coverage of 60% approximates what could be achieved long-term in tribal communities, where routine infant immunisation reaches 64% compared with 78% in the general population (Supplementary Table S1). At this coverage, deaths were reduced by 8%–64% as standalone effectiveness increased from 0 to 1. Meaningful mortality reductions from PrEP required optimistic assumptions: with 100% standalone PrEP effectiveness and 90% PrEP coverage, deaths fell to 25 (a 95% decline compared to no PrEP; Fig. 4A).

Healthcare-seeking was the dominant determinant of rabies mortality (Fig. 4B). At a healthcare-seeking probability of 0.70, projected deaths declined modestly from 1549 to 1503 as PrEP coverage increased from zero to 90%. By contrast, increasing healthcare-seeking from 0.70 to 0.95 at zero PrEP coverage reduced deaths by 1,117, more than 20 times the achievable benefit from maximum PrEP scale-up with the same level of healthcare-seeking. Increasing PrEP coverage neither offset poor healthcare access nor meaningfully improved outcomes when PEP access was already high.

Dog vaccination coverage in unowned dogs led to large, consistent reductions in mortality across all PrEP coverage levels (Fig. 4C). The marginal benefit of PrEP diminished progressively as dog vaccination coverage increased. Under zero-dog vaccination, increasing PrEP levels from 0 to 90% reduced deaths by only 13%. At 70% dog vaccination coverage, deaths were low (≤81) regardless of PrEP.

The relative benefit of PrEP was consistent across multipliers for the unowned dog population, reducing deaths by 10–14% regardless of the assumed population size (Fig. 4D). However, the multiplier itself was a far more powerful driver of mortality, with deaths ranging from 27 (multiplier 1×) to 438 (multiplier 5×) at zero PrEP use, dwarfing the effect of any PrEP scenario. This confirms that the limited marginal value of PrEP reflects a structural feature of the model rather than an artefact of the calibrated dog population estimate.

Taken together, these sensitivity analyses demonstrate that healthcare-seeking, dog population size and vaccination coverage were the dominant determinants of rabies mortality, substantially outweighing the influence of PrEP coverage. Improving PEP access and reducing rabies transmission through dog vaccination produced far greater mortality reductions than expanding routine childhood PrEP.

## Discussion

In Kerala, a setting with near-universal PEP access and high completion rates, routine childhood PrEP marginally reduces rabies mortality at substantial additional cost and was not optimal across India's WTP range. Sensitivity analyses further demonstrate that this conclusion is robust across a broad range of healthcare-seeking conditions, with strengthening dog vaccination and improving access to PEP consistently producing greater health gains than expanding routine childhood PrEP. By contrast, dog vaccination, particularly of unowned dogs, dramatically reduces mortality, with deaths approaching zero within three years under high coverage. Adopting the WHO's abridged 1-week PEP regimen is cost-saving with no loss of effectiveness. These findings hold even under the most optimistic assumptions about PrEP with dog vaccination averting three to four times as many deaths as PrEP at comparable cost.

A strength of our approach is dissecting how deaths are prevented, distinguishing contributions of MDV, PEP, and PrEP. Universal PrEP has been advocated for in India, arguing that PEP alone is insufficient and that children's disproportionate burden justifies a prophylactic approach.^27^ Our findings partially support the first premise: PEP cannot close the mortality gap, as demonstrated by Kerala's persistent deaths, but challenge the proposed solution. Recent analysis of laboratory-confirmed rabies cases in India found that only three out of 89 deaths who received PEP met the criteria for true vaccine failure; the remainder reflected delayed presentation, incomplete PEP, and absent RIG.^28^ Each vaccine delivery failure could be addressed by interrupting transmission in dogs, whereas PrEP would address very few. Advances in PEP biologicals offer welcome improvements in safety and tolerability,^29^ but alternate measures are required to address delays in care seeking and late presentations.

The dominant role of dog vaccination is consistent with established evidence that dogs are the primary reservoir from which other species are infected incidentally. In Kerala, cats account for approximately 40% of bite patients but fewer than 3% of rabies deaths, a disparity consistent with their characterisation as incidental vectors, not reservoirs.^30^ Where dog vaccination has eliminated rabies, exposures declined proportionally,^20^ suggesting that MDV scale-up would substantially reduce cat and wildlife exposures without additional feline or wildlife programmes.^31^ Scenario G (moderate vaccination of unowned dogs) ranks third by NHB, below Scenarios I and L, suggesting that modest reductions in catch-vaccinate-release costs through operational learning or improved delivery could shift it to the optimal strategy. Experience from Goa has demonstrated that achieving high-coverage MDV is feasible but requires coordinated vaccination teams, sustained workforce capacity, and continued investment.^32^ Future work should refine MDV costing estimates as programmes mature to better capture economies of scale, long-term workforce requirements, and the costs of sustaining high vaccination coverage. As Kerala shares borders with other rabies-endemic states, long-term success will also depend on regional coordination to minimise reintroduction.

PrEP may offer individual-level protection in tribal communities where structural barriers limit timely PEP access. However, the same barriers that constrain PEP are likely to constrain PrEP delivery, attenuating any population-level benefit. Sensitivity analyses show that even at tribal-level vaccination coverage (∼60%) and full standalone effectiveness, PrEP averted fewer deaths than moderate MDV scale-up, reinforcing that dog vaccination remains the priority intervention even in underserved subpopulations. As deaths approach zero, all strategies exhibit diminishing marginal returns, and the lower probability of optimality observed in near-zero scenarios should be interpreted accordingly.

The limited population-level impact of routine childhood PrEP reflects several interacting factors. Kerala already has high healthcare-seeking and PEP completion, leaving little room for additional benefit. Routine immunisation also builds coverage slowly, reaching only about 20% in ten years. Finally, the base-case model assumes no standalone protection from PrEP, consistent with WHO guidance. However, sensitivity analyses show that relaxing this assumption has only a modest quantitative effect and does not alter the overall conclusions, with dog vaccination remaining the preferred strategy across realistic coverage scenarios.

No scenario achieved zero cumulative deaths over ten years. However, under high-coverage dog vaccination, annual rabies deaths approached zero within approximately three years, indicating rapid interruption of transmission. The remaining cumulative deaths occurred predominantly before this point, while the small residual burden thereafter reflects the continued risk of rabies reintroduction from neighbouring areas and ongoing dog population turnover rather than a limitation of mass dog vaccination itself.

A critical finding is the likely underestimation of Kerala's unowned dog population. Our model required fivefold the census estimate of unowned dogs to reproduce surveillance data, yielding over two million total dogs, nearly three times the official figure. Planned dog vaccination campaigns may therefore fall short of the coverage needed to interrupt transmission. Field validation of Kerala's dog population should therefore be an operational priority. Kerala's human rabies surveillance has improved substantially in recent years, and our calibrated ascertainment estimate of 70% likely reflects real progress, making surveillance an increasingly reliable basis for monitoring programme impact.

Several limitations warrant acknowledgement. We model only direct health system expenditure, omitting costs borne by patients. These include travel, lost productivity, caregiver time, and the psychological burden of animal bites. Including them would further favour dog vaccination, which prevents bite exposures altogether and avoids both direct and indirect expenditure. Dog rabies incidence, biting rates and case–fatality parameters are drawn from Tanzanian data, the most comprehensive available, but may not fully translate to Kerala. In addition, health losses are estimated using the average age at rabies exposure and are not age stratified. Because rabies disproportionately affects children,^2^ this may underestimate the health benefits of interventions that prevent deaths, although we do not expect it to materially alter the relative ranking of strategies. Although we assume zero standalone PrEP effectiveness in the base case, sensitivity analyses confirm that this assumption does not alter our conclusions, as dog vaccination remains dominant across the full range of effectiveness. Whether PrEP uptake alters health-seeking behaviour remains uncertain: wider coverage could reduce perceived urgency to seek PEP, though proactively vaccinated individuals may be more health-seeking overall. Finally, our 10-year horizon may underestimate longer-term PrEP benefits as coverage builds across birth cohorts. However, the timescales to achieve meaningful coverage make PrEP a poor tool for near-term mortality reduction compared to dog vaccination, which can be scaled more quickly.

These findings carry clear policy implications for Kerala and other rabies-endemic settings. Immediate adoption of the abridged 1-week PEP regimen offers a cost-saving opportunity without requiring new infrastructure, but it would require revising India's national clinical guidelines and strengthening provider education and change–management activities to build confidence in the shorter regimen. Resources should then be prioritised towards strengthening dog vaccination, where achieving 70% coverage would reduce human rabies deaths to near zero within three years. The possibility that PrEP diverts resources from dog vaccination or PEP delivery must be treated as a risk, particularly given competition for programme management bandwidth and health budgets. Targeted PrEP may still have a role as an equity-focused intervention for specific high-risk, underserved populations. However, this should be supported through dedicated funding and implemented as a complement to, rather than a substitute for, core intervention strategies. The zero rabies by 2030 target requires sustained investment in dog vaccination, supported by equitable access to PEP, with PrEP reserved for carefully defined populations where its marginal benefit is greatest.

## Contributors

Conceptualisation: ZTN, KH, MML; Data curation: ZTN, HS, MA, RRJ, MML, EAF, KH; Formal analysis: MML, KH, EAF, DH, MA; Funding acquisition: ZTN, KH, MML; Investigation: MML, EAF, ZTN, DH, KH, RRJ, HS; Methodology: MML, EAF, ZTN, DH, KH, MA; Project administration: ZTN, KH; Resources: ZTN, KH, MML; Software: MML, EAF, KH, MA; Supervision: KH, ZTN, EAF, DH; Validation: KH, ZTN, EAF, DH, MA; Visualisation: MML, KH, DH, MA; Writing—original draft: MML; Writing—review & editing: MML, ZTN, DH, KH, MA. All authors have approved the final version. All authors have accessed and verified the data, and all authors were responsible for the decision to submit the manuscript.

## Data sharing statement

All analyses were performed in R version 4.6.0. Code, parameter tables, and supplementary analyses are available at https://github.com/RabiesResearch/PrEP_cost_effectiveness_ms.

## Declaration of interests

All authors declare no competing interests.
