# Supplementary material for: Pre-exposure prophylaxis as a complementary vaccination strategy to reduce rabies in Kerala, India: a cost-effectiveness modelling study
Source: Lancet Reg Health Southeast Asia. 2026 Sep 5;53:100854. doi: 10.1016/j.lansea.2026.100854 (PMC13571938; doi:10.1016/j.lansea.2026.100854)
Supplement: Supplementary file 1 [file mmc1.docx]

**Supplementary Tables & Figures**

**Supplementary Tables**

**Supplementary Table 1. Parameters for cost-effectiveness analysis of rabies prevention strategies.** Abbreviations: IDSP- Integrated Disease Surveillance Programme; QALYs – Quality-Adjusted Life Years; IBCM – Integrated Bite Case Management. *Government sources indicate data collected from informal interviews with key government employees. **Kerala's surveillance system captures a higher proportion of rabies deaths than national estimates (~10%); we assumed 50–70% ascertainment based on model calibration against recently reported deaths.

| **Parameter (p1-p21)** | **Value (general population)** | **Source** |
| --- | --- | --- |
| P1. Human population size being modelled | 35 million | Official projections^1^ |
| P2. Annual birth rate, expressed as a proportion of the population | 0·012 | Official projections^1^ |
| P3. Human-to-dog ratio, used for dog population estimates | 31(16) | Livestock census 2019^2^; calibrated |
| P4. Proportion of dogs that are unowned*,* and less accessible to vaccinate | 0·58 | Livestock census 2019^2^ baseline (0.257); calibrated |
| P5. Annual dog rabies incidence without dog vaccination | 0·0075-0·0125 | Literature^3,4^ |
| P6 & P7: Bite exposures per rabid dog. Negative binomial distribution with mean and dispersion parameters (*mu, k*). | *mu* 0·38, *k* 0·72 | Literature^3^ |
| P8. Probability that a person bitten by a rabid dog seeks healthcare. | 0·95 | IBCM pilot^5^ |
| P9. Annual incidence of animal bite victims presenting to health facilities per 1,000 population | 15·3 | IDSP data^6^ |
| P10. Probability that PEP is initiated following healthcare presentation | 0·9684 | IDSP data^6^ |
| P11. PEP adherence modelled as geometric decay with constant per-dose continuation probability (p = 0.965), calibrated to >90% completion of ≥3 doses | 0·965 | Community survey; IBCM pilot^5^ & calibrated |
| P12. Probability of death following rabies exposure in the absence of PEP | 0·17 | Literature^7^ |
| P13. Probability that incomplete PEP (<3 doses) prevents rabies | 0·986 | Literature^7^ |
| P14. Probability that complete PEP (≥3 doses if not PrEP’d) prevents rabies | 0·999 | Literature^7^ |
| P15. Proportion of infants with complete routine vaccination, , as a proxy for PrEP coverage | 0·778 (0·64 in tribal population) | National Family Health Survey^8^ |
| P16. Proportion of total population receiving PrEP during initial school-based campaign | 0·14 | Official projections^1^ |
| P17. Cost per vial of human rabies vaccine | $5 | Government sources* |
| P18. Average cost of RIG administration | $12 | Government sources* |
| P19. Cost per owned dog vaccinated at government clinics | $0·7-1·5 | Government sources* |
| P20. Cost per unowned dog vaccinated by catch–vaccinate–release | $4-5 | Government sources* |
| P21. QALYs gained per death averted (from Kerala’s life expectancy, 77 years, and average dog bite patient age (15 years) | 62 years | Literature^9,10^ |
| P22. Proportion of human rabies deaths recorded by surveillance | 0·5-0·7** | calibrated |

**Supplementary Table 2. Net health benefit, probability of optimality, and ranking of rabies prevention strategies across willingness-to-pay thresholds in Kerala, India (2026–2035).** See the cost-effectiveness plane and cost-effectiveness acceptability curves for these scenarios in Supplementary Figure 3.

|  | **Willingness-to-pay thresholds** | | | | | | | |
| --- | --- | --- | --- | --- | --- | --- | --- | --- |
| **Scenario** | US$200 | US$500 | US$1,000 | US$2,034 | US$2,535 | US$3,092 | US$4,000 | US$5,000 |
| A. SQ | 495,883 (14%, rank 3) | 646,668 (16%, rank 3) | 696,929 (11%, rank 4) | 722,480 (7%, rank 8) | 727,364 (6%, rank 8) | 730,935 (6%, rank 8) | 734,625 (5%, rank 8) | 737,139 (5%, rank 8) |
| B. SQ + PrEP (India regimen) | 438,586 (0%, rank 7) | 624,236 (3%, rank 7) | 686,119 (5%, rank 9) | 717,578 (4%, rank 10) | 723,591 (4%, rank 10) | 727,988 (4%, rank 10) | 732,532 (4%, rank 9) | 735,626 (4%, rank 9) |
| C. WHO PEP + 1-shot PrEP | 313,953 (0%, rank 12) | 574,326 (0%, rank 12) | 661,118 (0%, rank 12) | 705,239 (2%, rank 12) | 713,672 (2%, rank 12) | 719,839 (2%, rank 12) | 726,211 (3%, rank 12) | 730,551 (3%, rank 12) |
| D. WHO PEP & PrEP | 444,035 (0%, rank 6) | 626,238 (4%, rank 5) | 686,973 (5%, rank 7) | 717,847 (5%, rank 9) | 723,749 (5%, rank 9) | 728,065 (5%, rank 9) | 732,523 (5%, rank 10) | 735,560 (4%, rank 10) |
| E. D + drop in MDV | 446,921 (0%, rank 5) | 625,373 (4%, rank 6) | 684,857 (5%, rank 10) | 715,096 (4%, rank 11) | 720,876 (4%, rank 11) | 725,103 (4%, rank 11) | 729,470 (4%, rank 11) | 732,444 (4%, rank 11) |
| F. D + MDV scale-up in unowned dogs | 322,717 (0%, rank 11) | 588,736 (0%, rank 11) | 677,409 (2%, rank 11) | 722,487 (8%, rank 7) | 731,103 (9%, rank 6) | 737,404 (10%, rank 6) | 743,914 (11%, rank 6) | 748,348 (12%, rank 6) |
| G. SQ + moderate MDV in unowned dogs | 454,659 (1%, rank 4) | 639,203 (10%, rank 4) | 700,718 (14%, rank 3) | 731,990 (13%, rank 2) | 737,967 (13%, rank 3) | 742,338 (12%, rank 3) | 746,854 (12%, rank 3) | 749,930 (12%, rank 4) |
| H. SQ + MDV scale-up in unowned dogs | 374,638 (0%, rank 9) | 609,279 (1%, rank 9) | 687,492 (6%, rank 6) | 727,252 (10%, rank 4) | 734,852 (11%, rank 4) | 740,410 (12%, rank 4) | 746,152 (12%, rank 4) | 750,063 (13%, rank 2) |
| I. SQ + intense MDV in owned dogs | 502,352 (20%, rank 2) | 656,776 (28%, rank 2) | 708,251 (21%, rank 1) | 734,419 (16%, rank 1) | 739,420 (14%, rank 1) | 743,078 (13%, rank 2) | 746,857 (13%, rank 2) | 749,431 (12%, rank 5) |
| J. SQ + MDV scale-up in all dogs | 370,831 (0%, rank 10) | 607,822 (1%, rank 10) | 686,819 (5%, rank 8) | 726,977 (9%, rank 5) | 734,653 (10%, rank 5) | 740,267 (10%, rank 5) | 746,066 (11%, rank 5) | 750,016 (11%, rank 3) |
| K. WHO PEP + SQ MDV | 526,625 (64%, rank 1) | 658,612 (30%, rank 1) | 702,608 (16%, rank 2) | 724,973 (8%, rank 6) | 729,248 (7%, rank 7) | 732,374 (6%, rank 7) | 735,604 (6%, rank 7) | 737,804 (5%, rank 7) |
| L. WHO PEP + MDV scale-up all dogs | 403,010 (0%, rank 8) | 621,292 (3%, rank 8) | 694,053 (9%, rank 5) | 731,041 (14%, rank 3) | 738,111 (14%, rank 2) | 743,281 (15%, rank 1) | 748,623 (15%, rank 1) | 752,261 (15%, rank 1) |

**Supplementary Table 3. Net health benefit and probability of optimality of select rabies prevention strategies across willingness-to-pay thresholds in Kerala, India (2026–2035).** Figure 4 plots cost-effectiveness acceptability curves for these scenarios.

|  | **Willingness-to-pay thresholds** | | | | | | | |
| --- | --- | --- | --- | --- | --- | --- | --- | --- |
| **Scenarios** | US$200 | US$500 | US$1000 | US$2034 | US$2535 | US$3092 | US$4000 | US$5000 |
| A. SQ | 495,883 (15%) | 646,668 (21%) | 696,929 (18%) | 722,480 (15%) | 727,364 (15%) | 730,935 (15%) | 734,625 (14%) | 737,139 (14%) |
| B. SQ + PrEP (India regimen) | 438,586 (0%) | 624,236 (5%) | 686,119 (9%) | 717,578 (11%) | 723,591 (12%) | 727,988 (12%) | 732,532 (12%) | 735,626 (13%) |
| I. SQ + intense MDV in owned dogs | 502,352 (20%) | 656,776 (34%) | 708,251 (32%) | 734,419 (29%) | 739,420 (28%) | 743,078 (28%) | 746,857 (27%) | 749,431 (27%) |
| K. WHO PEP + SQ MDV | 526,625 (65%) | 658,612 (37%) | 702,608 (25%) | 724,973 (18%) | 729,248 (17%) | 732,374 (16%) | 735,604 (15%) | 737,804 (15%) |
| L. WHO PEP + MDV scale-up all dogs | 403,010 (0%) | 621,292 (4%) | 694,053 (16%) | 731,041 (26%) | 738,111 (28%) | 743,281 (29%) | 748,623 (31%) | 752,261 (32%) |

**Supplementary Figures**

**Supplementary Figure 1.** **Calibration of the decision-tree model across dog population size and healthcare-seeking probability.** Heatmap showing the composite loss function used to evaluate model fit across combinations of the unowned dog population multiplier (x-axis) and probability of healthcare seeking following a probable rabid exposure (y-axis). The composite loss equally weights deviations between model-predicted and observed rabies deaths and the proportion of bite patients classified as probable exposures. Shading indicates the parameter value's fitness. The highlighted point denotes the parameter combination selected for the base case, corresponding to the lowest composite loss

**Supplementary Figure 2. Projected rabies exposures, deaths, and programme costs under alternative intervention strategies in Kerala, India (2026–2035).** Lines show the median annual projections for A) rabies exposures, B) human rabies deaths, and C) total programme costs over a 10-year time horizon. Shaded ribbons represent 95 percentile intervals. Programme costs reflect combined expenditures for MDV, PEP, and PrEP across scenarios. Initial (year 1) costs are higher in PrEP scenarios due to a one-time school-based campaign, after which vaccination transitions to routine childhood delivery.

**Supplementary Figure 3. Cost-effectiveness across scenarios.** (A) cost-effectiveness plane comparing scenarios relative to the status quo. Each point represents a scenario, plotted by the incremental median number of deaths averted (x-axis) and incremental programme cost (y-axis). Circles denote strategies without pre-exposure prophylaxis (PrEP) and triangles denote strategies with PrEP. For PrEP scenarios, horizontal lines indicate the range of incremental deaths averted when the effectiveness of PrEP in the absence of subsequent post-exposure prophylaxis (PrEP effectiveness) is varied from 0 to 1; points represent the base-case assumption (PrEP effectiveness = 0). The solid black line shows the efficiency frontier after exclusion of strictly and extendedly dominated strategies. (B) Cost-effectiveness acceptability curves showing the probabilities that a strategy is optimal across a range of willingness-to-pay (WTP) thresholds. Vertical dashed lines indicate lower and upper bounds for India’s WTP (US$2,034 and US$3,092 per QALY gained, respectively). All costs are reported in 2026 US$. QALYs=quality-adjusted life-years. GDP=gross domestic product. WTP=willingness to pay.

**Supplementary Figure 4.** **Effect of reduced human rabies vaccine vial prices on the probability that routine childhood PrEP is the optimal strategy.** Cost-effectiveness acceptability curves comparing the base-case routine childhood PrEP strategy (Scenario B) with hypothetical reductions in human rabies vaccine vial price to 50% (B2) and 25% (B3) of the base-case value. The comparator set includes the status quo (A), WHO’s abridged one-week PEP regimen (K), intensive vaccination of owned dogs (I), and scale-up of vaccination in owned and unowned dogs (L). Vertical dashed lines indicate the lower and upper estimates of India’s willingness-to-pay threshold (US$2,034 and US$3,092 per QALY gained). All costs are reported in 2026 US$. QALYs=quality-adjusted life-years. GDP=gross domestic product. WTP=willingness to pay.
